# Pressure dependent half-metallic ferromagnetism in inverse Heusler alloy Fe_2_CoAl: a DFT+U calculations

**DOI:** 10.1039/d0ra07543d

**Published:** 2020-12-17

**Authors:** D. P. Rai, Lalrinkima, Lalhriatzuala, L. A. Fomin, I. V. Malikov, Adlane Sayede, Madhav Prasad Ghimire, R. K. Thapa, Lalthakimi Zadeng

**Affiliations:** Physical Sciences Research Center, Department of Physics, Pachhunga University College Aizawl 796001 India dibya@pucollege.edu.in; Department of Physics, Mizoram University Aizawl 796009 India; Institute of Microelectronics Technology and High Purity Materials RAS 142432 Chernogolovka Russia; Univ. Artois, CNRS, Centrale Lille, ENSCL, Univ. Lille, UMR 8181-UCCS-Unité de Catalyse et Chimie du Solide F-62300 Lens France; Central Department of Physics, Tribhuvan University Kathmandu Nepal madhav.ghimire@cdp.tu.edu.np

## Abstract

We report the electronic and magnetic properties along with the Curie temperature (*T*_C_) of the inverse full Heusler alloy (HA) Fe_2_CoAl obtained by using the first-principles computational method. Our calculations suggests that Fe_2_CoAl is a magnetic metal when treated within PBE-GGA under the applied compressive pressures. However, the implementation of electron–electron (U) (*i.e.*, GGA+U) with varying compressive pressure (*P*) drastically changes the profile of the electronic structure. The application of GGA+U along with pressure induces ferromagnetic half-metallicity with an integer value of total magnetic moment ∼4.0 *μ*_B_ per unit cell. The integer value is in accordance with the Slater–Pauling's rule. Here, we demonstrate the variation of semiconducting gap in the spin down channel. The band gap increases from 0.0 eV to 0.72 eV when increasing the pressure from 0 to 30 GPa. Beyond 30 GPa, the electronic band gap decreases, and it is completely diminished at 60 GPa, exhibiting metallic behaviour. The analysis of the computed results shows that the treatment of electron–electron interactions within GGA+U and the application of compressive pressure in Fe_2_CoAl enables d–d orbital hybridization giving rise to a half-metal ferromagnet. The *T*_C_ calculated from mean field approximation (MFA) decreases up to 30 GPa and then increases linearly up to 60 GPa.

## Abstract 2

We report the electronic and magnetic properties along with the Curie temperature (*T*_C_) of the inverse full Heusler alloy (HA) Fe_2_CoAl obtained using the first-principles computational method.

## Graphical abstract

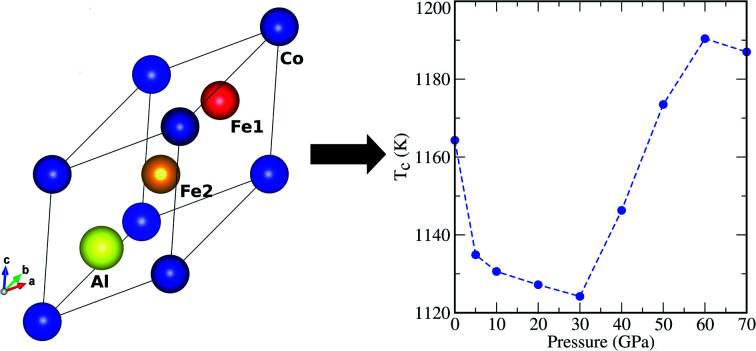

## 1 Introduction

The discovery of the prototype Heusler alloy Cu_2_MnAl in 1903 (ref. 1) initiated new research interest due to its ferromagnetic behaviour despite having all non-magnetic constituents.^1,2^ Heusler alloys have been studied for 120 years, but the application of these materials in spintronics has only been considered for 30–40 years.^3–12^ Half-metal ferromagnetism (HMF) is the top priority among all the explored properties of Heusler alloys.^13–20^ HMF occurs when magnetic materials exhibit metallic behavior in one of the spin channels whereas the other spin channel is semiconducting. Rigorous research has been performed in order to understand the underlying mechanism of unusual ferromagnetic behaviour in terms of atomistic scale interactions. Several theories and models have been proposed for the integration of these exotic properties in new technological applications in spintronics.^21^ Spintronics is a branch of science which deals with the charge and spin of an electron. High capacity storage devices, magnetic RAM, spin-injection, spin-valves, spin-filters, GMR, TMR, and many more potential electron spin-based components are foreseen.^22–25^ To improve the storage capacity of memory devices, the manipulation of spin degrees of freedom is crucial. Fabrication of practical devices with enhanced efficiency is another challenging task. Some of the Heusler alloys with high spin polarization and high Curie temperature^21,26^ are promising and may complement this goal. Although the practical application of HMF in spintronic devices is in the preliminary stage, lab work is still in progress. Heusler alloys with 3d-orbitals are very sensitive to externally applied fields (temperature, pressure, electric and magnetic fields), and as a result their electronic and magnetic properties can be tuned very readily. The d–d orbital hybridization between the transition metals is reported to be responsible for some of their outstanding electronic and magnetic properties, including the high value of spin polarization at the Fermi level (*E*_F_).^18^ They exhibit low Gilbert damping, high Curie temperature, high spin polarization, *etc.*^27–37^ In addition, Heusler alloys are also explored as potential thermoelectric materials^38^ due to the presence of non-toxic components and their large range of working temperatures as compared to other thermoelectric materials.^39–42^ Currently, numerous theoretical and experimental research investigations are in progress to study the electronic and magnetic properties of Heusler alloys *via* structural modifications under applied strain and hydrostatic pressure. Under applied hydrostatic pressure, the bond lengths vary due to the displacement of atoms from their mean positions and changes electronic charge densities which have a direct impact on the electronic and magnetic properties.^43,44^ Ram *et al.* have reported significant changes in the band structure of Co_2_XY (X = Cr, Mn and Y = Al, Ga) type direct full-Heusler alloys under applied pressure.^45^ Rasul *et al.*^46^ have studied the quaternary Heusler alloys ScNiCrX (X = Al, Ga) using a first-principles approach and reported the robustness of the half-metallicity up to 6 GPa and 16 GPa for ScNiCrAl and ScNiCrGa, respectively. Amudhavalli *et al.*^47^ studied Fe-based ferromagnetic quaternary Heusler alloys and reported a pressure induced structural phase transition at 151.6 GPa, 33.7 GPa, 76.4 GPa, 85.3 GPa, 87.7 GPa and 96.5 GPa for CoFeTiSi, CoFeTiGe, CoFeTiAs, NiFeTiSi, NiFeTiGe and NiFeTiAs, respectively. The same group,^48^ performing similar kinds of studies, have reported a half-metal to metal phase transition for Co_2_TiAl, Co_2_TiGa and Co_2_TiIn under applied external pressures of 76.5 GPa, 73.1 GPa and 63.9 GPa, respectively. Rambabu *et al.* studied the variation of the Curie temperature (*T*_C_) under applied pressure and reported enhanced *T*_C_ at high compressive pressures.^49^ There are reports on the variation of *T*_C_ of various Heusler alloys (such as Ni–Mn based alloys) under applied pressure.^50–52^ Dannenberg *et al.*^53^ reported the structural dependence of *T*_C_ of Fe_2_CoGa: ∼780 K for L2_1_ and ∼770 K for L1_0_, respectively. Some more promising results for *T*_C_s for Fe-based full-Heusler alloys^54^ under ambient conditions are: 925 K (Fe_2_CoGe), 750 K (Fe_2_NiGe), 845 K (Fe_2_NiGa), 798 K (Fe_2_CuGa), and 875 K (Fe_2_CuAl). In our previous work^55^ only Co_2_FeAl exhibited half-metallicity with the implementation of DFT+U whereas Fe_2_CoAl was metallic. However, there is a pseudo bandgap above the Fermi energy in the spin-down channel of Fe_2_CoAl within DFT+U. Thus we were encouraged to implement external pressure to observe the location of the bandgap. Interestingly, we have observed a projection of the bandgap in the Fermi level in the spin-down channel, giving rise to half-metallicity. Therefore, we have applied pressure, which shortens the bond lengths and facilitates d–d hybridization, which may give the desired results. In this paper, we have also tried to explore the electronic and magnetic properties along with the *T*_C_ under compressive pressure using the first-principles approach. We report the sensitiveness of the electronic and magnetic properties under lattice strain due to applied pressure.

## 2 Computational details

**Fig. 1:**
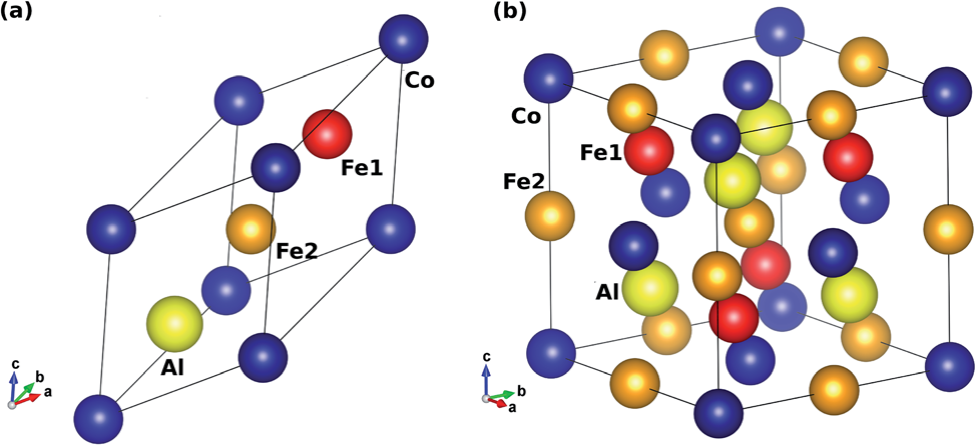
(a) Primitive and (b) conventional unit cell of the inverse Heusler alloy Fe_2_CoAl with space group *F*4̄3*m*. Balls in red, orange, blue and yellow in the represents Fe1, Fe2, Co and Al respectively.

In terms of their chemical compositions, Heusler alloys are classified as binary (X_3_Z), ternary (X_2_YZ or XYZ), and quaternary (AXYZ) compounds, where A, X and Y are transition metals and Z is a p-block element. Binary HAs only have two distinguishable elements in the unit cell. In general, ternary HAs are of two types *viz.* full (X_2_YZ) and half (XYZ) HAs. The stoichiometric atomic ratios of full (X_2_YZ) and half (XYZ) ternary HAs are 2 : 1 : 1 and 1 : 1 : 1, respectively. On the other hand, quaternary HAs have an equiatomic stoichiometric ratio of 1 : 1 : 1 : 1. Herein, we focus on ternary full-HAs. Ternary full-HAs crystallize in two ordered phases: Cu_2_MnAl-type with space group *Fm*3̄*m* (225)^35,56^ and Hg_2_CuTi-type with the *F*4̄3*m* (216) space group.^57,58^ The former is referred to as regular/direct (L2_1_) and the latter is inverse/indirect with XA-structure. The atomic Wyckoff positions for both the XA-phase and the L2_1_-phase of Fe_2_CoAl are tabulated in Table 1. In the case of ternary inverse full-HAs the electronegativity of the X-atom is less than that of the Y-atom (X = Fe and Y = Co). Thus, our Fe_2_CoAl system is an inverse full-HA with the electronegativity of the Fe-atom being less than that of the Co-atom. The primitive and conventional unit cells of Fe_2_CoAl with XA-structure are shown in Fig. 1. To study the electronic and magnetic properties of the inverse full-Heusler alloy Fe_2_CoAl, we have performed first-principles Density Functional Theory (DFT) calculations.^59,60^ All electron–electron interactions were considered according to the generalized gradient approximation (GGA) within the Perdew–Burke–Ernzerhof (PBE) parametrization.^61^ We are aware that GGA is inadequate for treating strongly correlated d-electrons to derive accurate electronic structures. On the other hand, the successful obtainment of accurate electronic properties by using GGA+U has already been reported for many Heusler alloys.^62–67^ Therefore, we have applied a screened Coulomb interaction along with the conventional GGA (GGA+U or DFT+U) in order to deal with strongly correlated 3d electrons.^68^ We have adopted *U* parameters of 3.82 eV for Fe and 3.89 eV for Co according to our previous work.^55,69^ For computation, we have used the all electron orbital based full potential linearized augmented plane wave (FP-LAPW) basis set formalism as implemented in the WIEN2K package.^70^ The non-spherical cut off value of angular momentum within the muffin tin (MT) sphere is *l*_max_ = 10. *R*_MT_ × *K*_max_ = 7 where *K*_max_ is the maximum value of the reciprocal lattice vector in the plane wave expansion and *R*_MT_ is the smallest muffin tin (MT) radius. To model the electronic structure, the first Brillouin zone (BZ) was integrated by taking a 10 × 10 × 10 *k*-mesh grid within the Monkhorst package. The self-consistency convergence criterion was set to be 0.0001 Ry. The ferromagnetic–antiferromagnetic exchange energy *J*_ij_ is a key parameter in determining the Curie temperature (*T*_C_). A code based on the single-site coherent potential approximation within the Green’s function approach called spin-polarized relativistic Korringa–Kohn–Rostoker (SPR-KKR)^71,72^ was used for the calculation of *T*_C_.

**Table 1:** Wyckoff positions of the Fe_2_-based inverse full-Heusler alloy Fe_2_CoAl

Positions	*x*	*y*	*z*
**Inverse**			
Fe1	0.75	0.75	0.75
Fe2	0.50	0.50	0.50
Co	0.25	0.25	0.25
Al	0.00	0.00	0.00

**Direct**			
Fe1	0.25	0.25	0.25
Fe2	0.75	0.75	0.75
Co	0.50	0.50	0.50
Al	0.00	0.00	0.00

## 3 Results and discussion

### 3.1 Structural properties

**Fig. 2:**
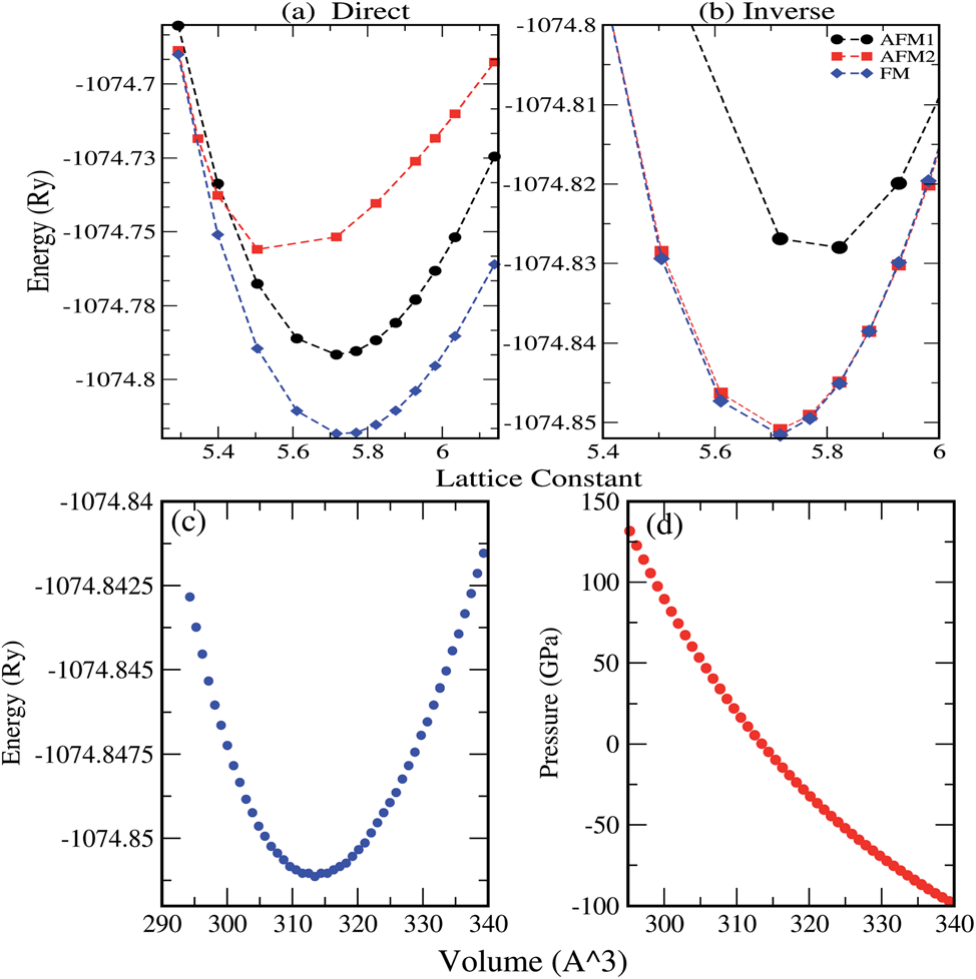
Variation of total energy as a function of lattice constant *a* (Å): (a) direct phase, (b) inverse phase (AMF1, AFM2 and FM configurations are represented by black, red and blue lines, respectively), (c) ground state energy as a function of volume in Å^3^ for FM and (d) pressure in GPa as a function of volume in Å^3^.

Our first-principles calculations start with the optimization of both the L2_1_ (direct) and XA (inverse) structures with different magnetic configurations. The initial magnetic configurations set for our calculations are ferromagnetic FM (Fe1↑, Fe2↑, Co↑), antiferromagnetic AFM1 (Fe1↑, Fe2↓, Co↑), and AFM2 (Fe1↑, Fe2↑, Co↓) (see Table 2). The variations in total energy *versus* the lattice constant *a* (Å) for both the direct and inverse phases with the FM, AFM1 and AFM2 magnetic configurations are shown in Fig. 2a and b. The XA-structure (inverse) with the FM configuration is energetically favourable with the minimum energy as shown by the blue line and the diamonds [see Fig. 2b]. The ground state energy and pressure as a function of volume are also presented in Fig. 2c and d. The calculated lattice constant is 5.73 Å and is consistent with previously reported values of 5.70 Å,^73^ 5.71 Å,^74^ 5.766 ± 0.05 Å,^75^ and 5.732 Å.^76^ This result also agrees well with the results for several other analogous Fe-based inverse Heusler alloys whose lattice parameters vary from 5.5 to 6.2 Å.^35,54,55,77–84^ Further, the cohesive energy has been calculated from eqn (1). The cohesive energy results are presented in Fig. 7c and confirm the ground state stability of each system under different applied pressures.1
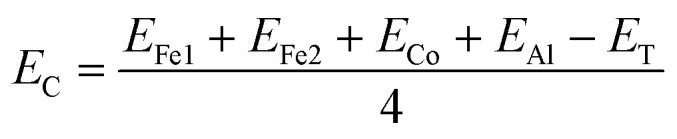
Here in eqn (1), *E*_Fe1_, *E*_Fe2_, *E*_Co_, *E*_Al_ and *E*_T_ are the individual energies of Fe1, Fe2, Co, and Al, and the total energy of the system, respectively. *n* = 4 denotes the total number of atoms in the unit cell.

**Table 2:** Magnetic configuration (MC) of the individual atoms Fe1, Fe2, Co, and Al, and total ground state energy *E*_T_ in Ry

MC	Fe1	Fe2	Co	Al	*E*_T_
**Inverse**					
FM	↑	↑	↑	N	−1074.852
AFM1	↑	↓	↑	N	−1074.829
AFM2	↑	↑	↓	N	−1074.8509

**Direct**					
FM	↑	↑	↑	N	−1074.818
AFM1	↑	↓	↑	N	−1074.792
AFM2	↑	↑	↓	N	−1074.758

### 3.2 Electronic and magnetic properties

**Fig. 3:**
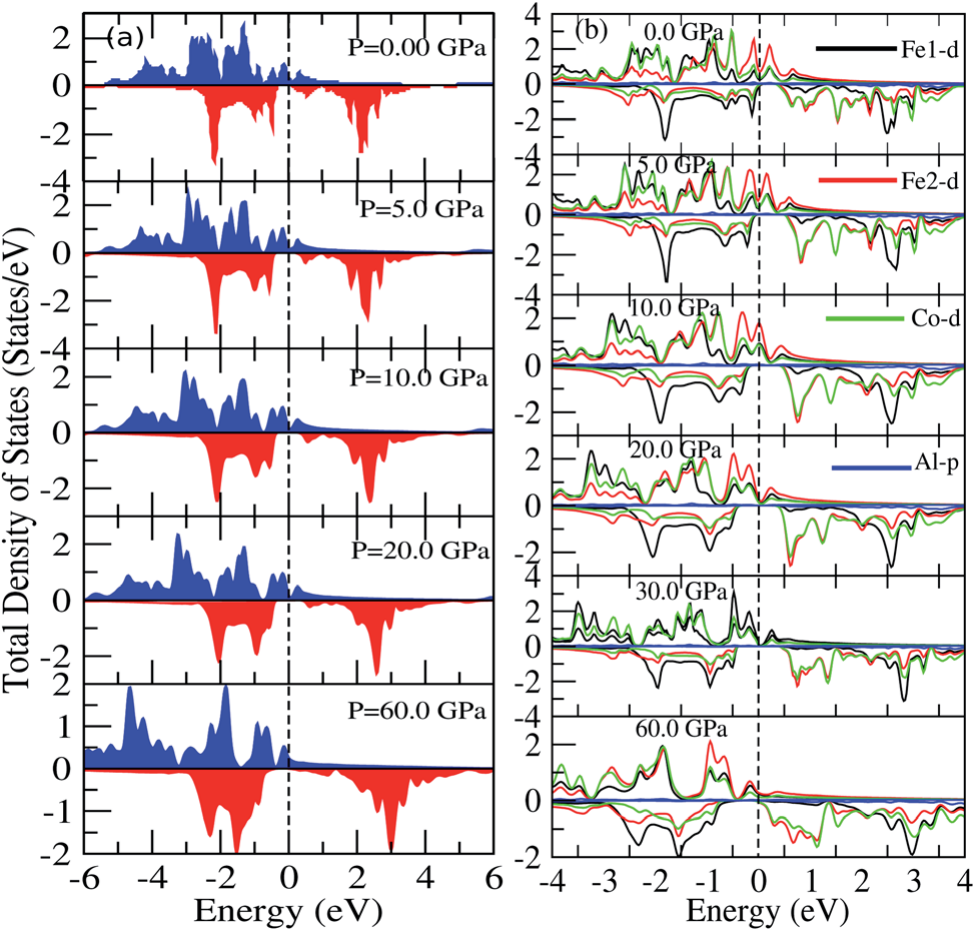
(a) Total DOS calculated using GGA+U, (b) partial DOS of Fe1-d, Fe2-d, Co-d and Al-p calculated with GGA+U at different pressures.

**Fig. 4:**
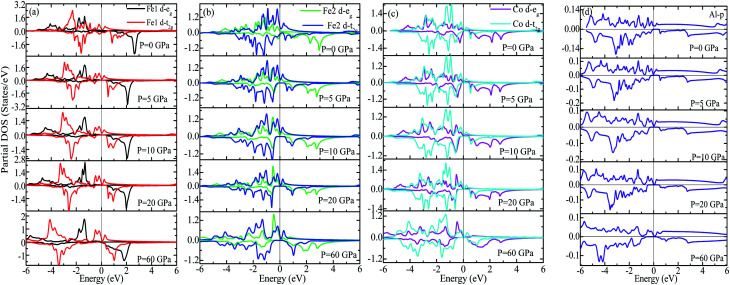
(a) Partial DOS of Fe1 (d-e_g_, d-t_2g_), (b) partial DOS of Fe2 (d-e_g_, d-t_2g_), (c) partial DOS of Co (d-e_g_, d-t_2g_) and (d) partial DOS of Al-p at different pressures.

**Fig. 5:**
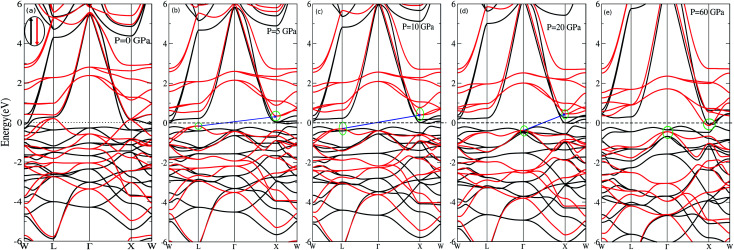
Band energies of Fe_2_CoAl calculated with GGA+U at (a) *P* = 0 GPa, (b) *P* = 5 GPa, (c) *P* = 10 GPa, (d) *P* = 20 GPa and (e) *P* = 60 GPa.

**Fig. 6:**
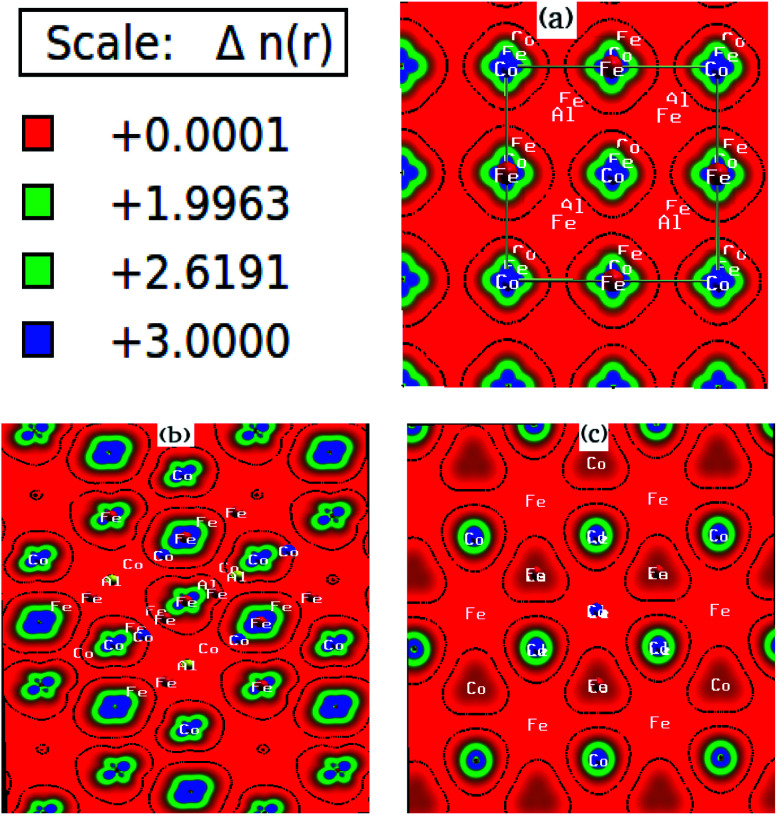
Electron density (e Å^−3^) of Fe_2_CoAl along the (a) 001, (b) 011 and (c) 111 directions.

**Fig. 7:**
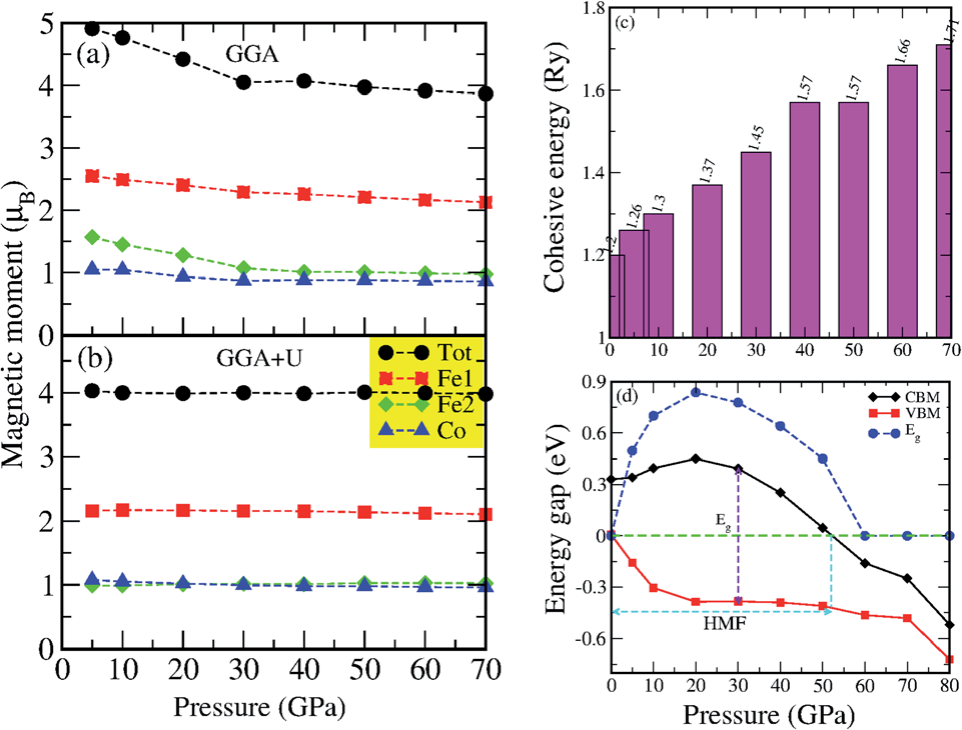
(a and b) Total and partial magnetic moments calculated with the FPLAPW method using WIEN2K, variation of (c) cohesive energy and (d) energy band gap as a function of pressure.

We have investigated the electronic properties of Fe_2_CoAl by calculating the total density of states (TDOS) and energy bands at different pressures using GGA+U [*cf.*Fig. 3–5]. We have already reported the inadequacy of GGA in deriving the electronic properties in our previous work.^55^ On the other hand, GGA+U has predicted a band gap in the spin down channel but this is well above the Fermi level (*E*_F_). We have observed the presence of some band edges at the *E*_F_, mostly originating from Fe1-d (t_2g_), Fe2-d (e_g_, t_2g_) and Co-d (e_g_, t_2g_) [*cf.*Fig. 4a–d]. However, the Fe1-d (e_g_) state is hardly seen in the picture as it lies far below the *E*_F_ in the spin up channel [*cf.*Fig. 4a]. Hence, we have proceeded with our calculations by using GGA+U along with the application of compressive pressure. Interestingly, on the application of compressive pressure (5 GPa), the *E*_F_ is pushed upward within the band gap [Fig. 3a and b]. The half-metallic band gap is attributed to the d–d hybridization between Fe2-d and Co-d followed by Fe1-d states to give bonding–antibonding states as in the case of other full-Heusler alloys.^15–19,26^ The bonding states at the top of the valence band form the valence band maximum (VBM) and the lowest antibonding states in the conduction region form the conduction band minimum (CBM). On the other hand, the spin up channel is still conducting. This hybrid characteristic of being semiconducting in the spin down channel and conducting in the spin up channel results in peculiar half-metal ferromagnetic (HMF) behaviour. We have also noticed widening of the band gap with increasing compressive pressure, as this facilitates hybridization due to shortening of the bond lengths. The energy band gap increases from 0.0 eV to 0.72 eV on increasing the pressure from 0 to 30 GPa [*cf.*Fig. 7d]. This result can also be confirmed by the energy band structure which exhibits the indirect nature of the band gap as measured along the L–X symmetry and denoted by the green circles [*cf.*Fig. 5]. In Fig. 3 and 4, we observe a large band gap of ∼0.72 eV at 30 GPa with the *E*_F_ pinned exactly in the middle of the band gap, providing more evidence of half-metallicity. A further increase in applied pressure (say beyond 30 GPa) decreases the band gap with drifting of the upper band edge (CBM) towards lower energy (specifically towards the *E*_F_). This can be seen in Fig. 3b and 4a–d in which the Fe1-d state has moved towards higher energy in the conduction band, taking part less in the d–d hybridization, and the coupled Fe2-d–Co-d bands are pushed towards lower energy (*i.e.* towards the *E*_F_) in the spin down region. It looks like the *E*_F_ shifts from the lower to the upper edge of the half-metallic band gap in the spin down channel on increasing the pressure, a typical feature of band flip. At 60 GPa the CBM (upper edge of the band gap) appears at the *E*_F_, thus diminishing the half-metallic behaviour and increasing the metallicity. The robustness of the half-metallicty is measured in terms of spin polarization at the *E*_F_. The degree of spin polarization in the vicinity of the *E*_F_ can be analyzed by *P* = [*N*_↑_(*E*_F_) − *N*_↓_(*E*_F_)]/[*N*_↑_(*E*_F_) + *N*_↓_(*E*_F_)], where *N*_↑_(*E*_F_) and *N*_↓_(*E*_F_) are the numbers of states at *E*_F_ for the spin up and spin down channels, respectively. Our GGA+U calculation with pressure has significantly improved the spin polarization by more than 45%. At 5 GPa we estimated ∼98% spin polarization. On varying the pressure, 5 < *P* < 60 GPa, we have achieved perfect half-metallic behaviour in our Fe_2_CoAl system. The projection of *E*_F_ inside the band gap in the spin down channel and the finite value of the electron density around *E*_F_ in the spin up channel at 5 < *P* < 60 GPa results in 100% spin polarization. To analyse the nature of the bonding between the atoms we have calculated the valence electron charge density (e Å^−3^) and the results at 30 GPa are presented in Fig. 6. The analysis of the charge density shows the presence of metallic bonding between the atoms and no sign of covalent bonds.

As reported elsewhere, the magnetic properties of perfect half-metallic ferromagnet (HMF) Heusler alloys (HAs) can be predicted from the total number of valence electrons present in the unit cell.^15–18,26^ The total magnetic moment can be derived from the Slater–Pauling (SP) rule^18^ as given by *M*_t_ = (*Z*_t_ − 24) *μ*_B_, where *M*_t_ is the total magnetic moment and *Z*_t_ is the total number of valence electrons. The total number of valence electrons in our Fe_2_CoAl system is 2 × 8 + 9 + 3 = 28. So the expected value of the total magnetic moment is *M*_t_ = 4.0 *μ*_B_ which results in the half-metallicity. The total magnetic moment obtained from GGA sharply deviates from the Slater–Pauling rule. However, on treating the system within GGA+U and applying pressures (0–70 GPa), the total magnetic moment *M*_t_ varies around 4.0 *μ*_B_. The highest *M*_t_ = 4.440 *μ*_B_ and the lowest *M*_t_ = 3.981 *μ*_B_, calculated at 0 GPa and 70 GPa, respectively [Table 3]. The *M*_t_ values at 0 and 70 GPa do not comply with the SP rule. Also, at 60 GPa *M*_t_ = 3.988 *μ*_B_ and Δ*μ*_B_ = ∼0.012 or ∼0.3%. At applied pressures of 5 < *P* < 60 GPa, the *M*_t_ value is ∼4 *μ*_B_. Thus, we can claim that the *M*_t_ values are in accordance with the SP rule^18^ at 5 < *P* < 60 GPa. We also present the variation of the partial magnetic moments calculated with GGA and GGA+U under different pressures in Fig. 7a and their numerical values are tabulated in Table 3.

**Table 3:** Total and partial magnetic moments (in *μ*_B_) calculated with GGA+U along with the Curie temperature *T*_C_ (in K)

*P* (GPa)	*M*_Tot_	*M*_Fe1_	*M*_Fe2_	*M*_Co_	*T* ^cal^_C_	*T* ^MFA^_C_
0.0	4.440	2.126	1.011	1.109	826.640	1164.30
5.0	4.030	2.158	0.989	1.075	752.430	1134.90
10.0	3.999	2.171	0.990	1.051	746.819	1130.60
20.0	3.990	2.165	1.010	1.018	745.190	1127.20
30.0	4.000	2.155	1.016	0.995	747.000	1124.20
40.0	3.996	2.152	1.009	0.979	744.828	1146.30
50.0	4.009	2.137	1.025	0.978	748.629	1173.50
60.0	3.988	2.121	1.028	0.964	746.276	1190.40
70.0	3.981	2.105	1.024	0.957	743.561	1187.00

**Fig. 8:**
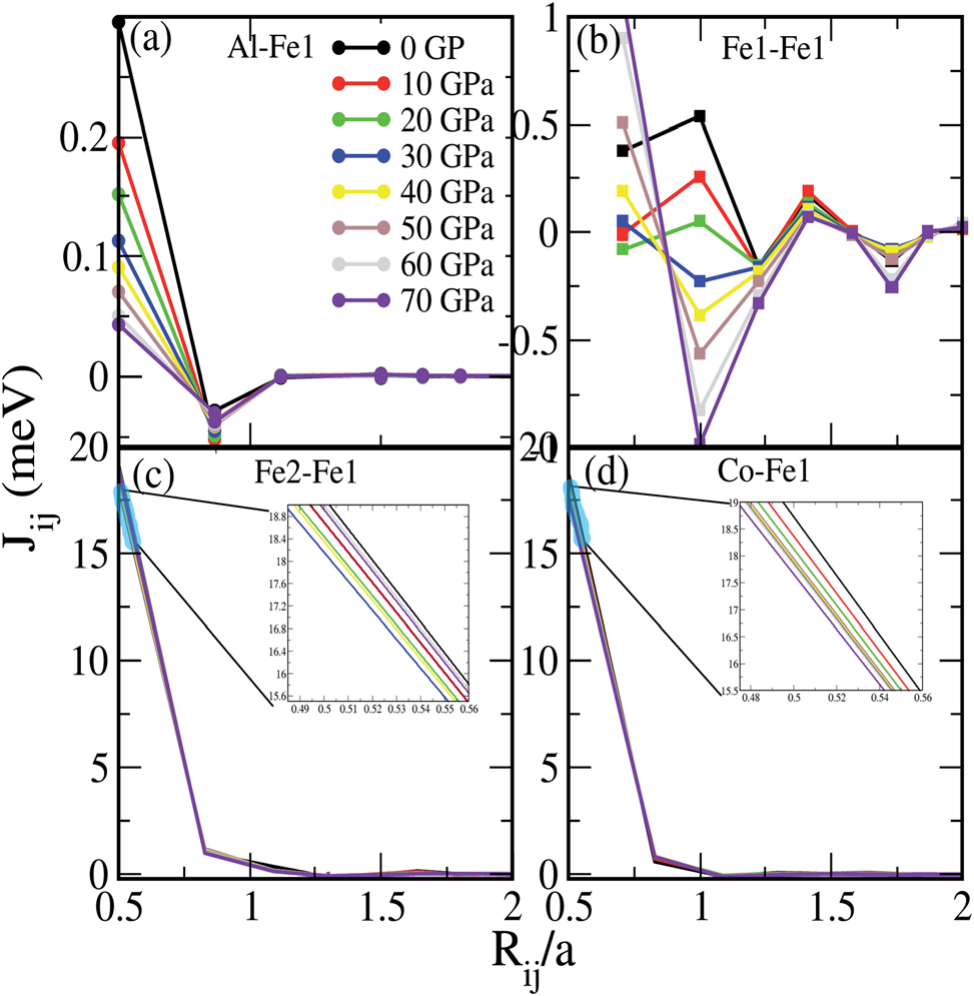
Exchange interaction *J*_ij_ (meV) between (a) Al and Fe1, (b) Fe1 and Fe1, (c) Fe2 and Fe1 and (d) Co and Fe1 at different pressures.

**Fig. 9:**
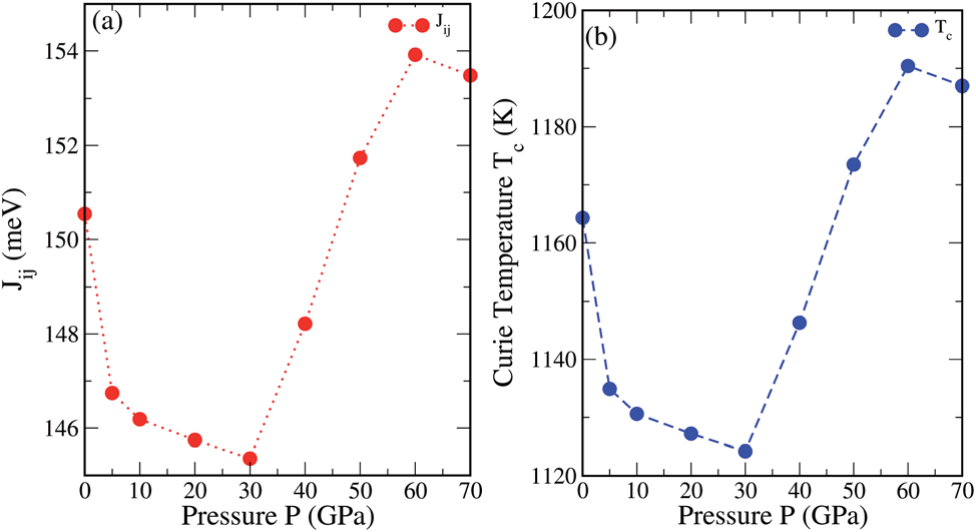
(a) Exchange interaction *J*_ij_ (meV) and (b) Curie temperature *T*^MFA^_C_ (K) as a function of pressure.

In order to further understand the magnetic interactions and magnetic properties, we have calculated the magnetic exchange energy by modeling the pair exchange interaction parameter *J*_ij_. The *J*_ij_ parameter is computed using the Heisenberg model.^17^ The exchange coupling interactions are mapped between the atoms sitting at the i and j sites separated by some distance as in eqn (2)2
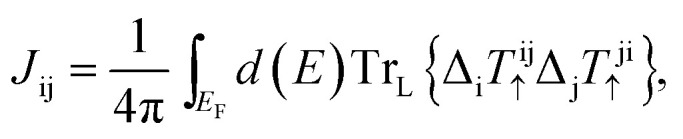
where Δ_i_ = *t*_i↑_^−1^ − *t*_j↓_^−1^, *t*_↑↓_^−1^ is the atomic *t*-matrix of the magnetic impurities at site i for the spin up/down state, *T*^ij^_↑↓_ is the scattering path operator between the i and j sites for the spin up/down state, and Tr_L_ is the trace over the orbital variables of the scattering matrices. The calculated exchange parameters *J*_ij_ for a central Fe1 atom interacting with all other atoms (Al–Fe1, Fe1–Fe1, Fe2–Fe1 and Co–Fe1) as a function of *R*_ij_/*a* at different pressures are shown in Fig. 8a–d. The estimated *J*_ij_ is a key parameter for obtaining the Curie temperature (*T*_C_) in the mean-field approximation (MFA) as given in eqn (3):3
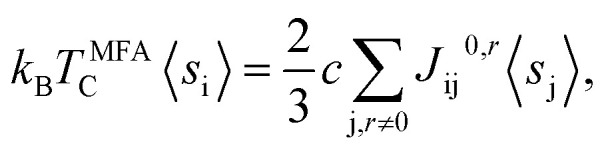
where *k*_B_ is the Boltzmann constant, *c* is the concentration of the impurities, and 〈*s*_j_〉 is the average j component of the unit vector *s*_*r*_^j^ along the direction of magnetization. The *J*_ij_ below *R*_ij_/*a* = ∼1 (below 1) shows a finite stable value. Meanwhile, the *J*_ij_ values above *R*_ij_/*a* = ∼1 (above 1) either remain close to 0 meV or fluctuate around 0 meV [*cf.*Fig. 8a–d]. The variation of the total *J*_ij_ along with the calculated *T*^MFA^_C_ as a function of *R*_ij_/*a* under different pressures are shown in Fig. 9a and b. We can see that on increasing the pressure up to 30 GPa the *J*_ij_ parameter decreases systematically. This leads to a decrease in *T*^MFA^_C_. As shown in Fig. 8c the *J*_ij_ value is governed by a strong interaction between Fe1 and Fe2. The lowest calculated value of *T*^MFA^_C_ = 1124.20 K at 30 GPa is mainly attributed to the low value of *J*_ij_ due to the short range (*R*_ij_/*a* < ∼1) interaction between Fe1 and Fe2 [see inset (blue line) in Fig. 8c]. For the Al–Fe1, Fe1–Fe1 and Co–Fe1 interactions, the *J*_ij_ values are intermediate at 30 GPa [*cf.*Fig. 8a–d]. On increasing the pressure beyond 30 GPa there occurs a linear increase in *T*^MFA^_C_, which reaches a maximum at 60 GPa. Our results for *T*^MFA^_C_ contradict with the results of Rambabu *et al.* for Co_2_CrX (X = Al, Ga, In) below 30 GPa, whereas at high pressure, *i.e.*, above 30 GPa they follow a similar trend.^49^ The calculated *T*_C_ values obtained from the mean field approximation are tabulated in Table 3. The other method to estimate the *T*_C_ in relation to the total magnetic moment (*M*_t_) of HMF-HAs is given by eqn (4).^19,26^

4 *T*^cal^_C_ = 23 + 181 × *M*_t_

As we have already discussed the inefficiency of GGA in deriving the half-metallicity, estimating *T*_C_ by taking the *M*_t_ obtained from GGA is not justifiable. Therefore, we have taken the *M*_t_ values calculated with GGA+U and substituted in eqn (4) to obtain the *T*^cal^_C_. The estimated values of *T*^cal^_C_ are presented in Table 3. The *T*^cal^_C_ values vary from 826.640 K to 743.561 K on varying the pressure from 0 to 70 GPa. These results are in good agreement with the *T*_C_s of other analogous Fe-based inverse full-HAs.^54^ We have noted that the *T*_C_ obtained from eqn (4) appears to be independent of interaction strength as the variation of the total magnetic moment (*M*_t_) is very small. Referring to Table 3, the *T*_C_ values calculated with the MFA (eqn (3)) look much higher as compared to those calculated with eqn (4). The large values of *T*^MFA^_C_ may arise due to inability to include the magnetic percolation effect within the mean field approximation.^55^

## 4 Conclusion

In summary, we have studied the structural, electronic and magnetic properties of the inverse HA Fe_2_CoAl along with the *T*_C_ values obtained from GGA and GGA+U under applied pressures. We have shown that the strong correlation mainly comes from the Fe-3d and Co-d states, and the inclusion of electron–electron interactions within GGA as the GGA+U formalism is essential to describe the electronic properties. The implementation of GGA+U along with compressive pressure (5 < *P* < 60 GPa) leads to half-metallic behavior with the opening of a spin minority band gap. The predicted integer value of the total magnetic moment in the inverse full-Heusler alloy Fe_2_CoAl, ∼4.0 *μ*_B_ at 30 GPa, is in accordance with the Slater–Pauling rule, which supports the half-metallicity. The *T*_C_ calculated from eqn (4) is 747 K at 30 GPa, in good agreement with the results of other Fe-based inverse full-HAs. However, the results from MFA are overestimated.

## Conflicts of interest

There are no conflicts to declare.

## Supplementary Material
